# Identifying Two New Ros/MucR Proteins: An Atypical Structure with a Divergent Function

**DOI:** 10.3390/biom16060781

**Published:** 2026-05-26

**Authors:** Domenico Sgambati, Ilaria Imperatrice, Enza Canonico, Rosita Russo, Martina Slapakova, Maria Diletta Cinque, Luciano Pirone, Gianluca D’Abrosca, Sahiba Gul, Martina Dragone, Isabella Maria Acquistapace, Antonio Chaves-Sanjuan, Haidi Shehi, Diane Marie Valérie Bonnet, Daniel Pérez-Mendoza, Remus Thei Dame, Carla Isernia, Roberto Fattorusso, Luigi Russo, Gaetano Malgieri, Marco Nardini, Juan Sanjuan, Angela Chambery, Emilia Maria Pedone, Paolo Vincenzo Pedone, Ilaria Baglivo

**Affiliations:** 1 Department of Environmental, Biological, Pharmaceutical Sciences and Technologies, University of Campania “Luigi Vanvitelli”, Via Vivaldi 43, 81100 Caserta, Italy; domenico.sgambati@unicampania.it (D.S.); ilaria.imperatrice@unicampania.it (I.I.); enza.canonico@unicampania.it (E.C.); rosita.russo@unicampania.it (R.R.); martina.slapakova@unicampania.it (M.S.); sahiba.gul@unicampania.it (S.G.); martina.dragone@unicampania.it (M.D.); carla.isernia@unicampania.it (C.I.); roberto.fattorusso@unicampania.it (R.F.); luigi.russo2@unicampania.it (L.R.); gaetao.malgieri@unicampania.it (G.M.); angela.chambery@unicampania.it (A.C.); 2 CNR—Institute of Biostructures and Bioimaging, Via Pietro Castellino 111, 80131 Naples, Italy; mariadilettacinque@cnr.it (M.D.C.); luciano.pirone@cnr.it (L.P.); emilia.pedone@cnr.it (E.M.P.); 3 Department of Human Sciences, Link Campus University, Via del Casale di San Pio V 44, 00165 Roma, Italy; gianluca.dabrosca@unicampania.it; 4 Department of Biosciences, University of Milan, Via Celoria 26, 20133 Milano, Italy; isabella.acquistapace@unimi.it (I.M.A.); antonio.chaves@unimi.it (A.C.-S.); haidi.shehi@unimi.it (H.S.); diane.bonnet@unimi.it (D.M.V.B.); marco.nardini@unimi.it (M.N.); 5 Fondazione Romeo e Enrica Invernizzi and NOLIMITS, University of Milan, Via Celoria 26, 20133 Milan, Italy; 6 Department of Soil and Plant Microbiology, Estación Experimental del Zaidín, CSIC, 18008 Granada, Spain; dpmendoza@eez.csic.es (D.P.-M.); juan.sanjuan@eez.csic.es (J.S.); 7 Macromolecular Biochemistry, Leiden Institute of Chemistry, Leiden University, 2333 CC Leiden, The Netherlands; 8 Centre for Microbial Cell Biology, Leiden University, 2333 CC Leiden, The Netherlands; 9 Centre for Interdisciplinary Genome Research, Leiden University, 2333 CC Leiden, The Netherlands

**Keywords:** Ros/MucR family, H-NS-like proteins, protein–DNA interaction

## Abstract

The Ros/MucR family is constituted by proteins controlling the expression of genes crucial for the interaction with eukaryotic hosts. Ros/MucR family members were classified as H-NS-like proteins in α-proteobacteria, as they share fundamental features with H-NS proteins playing a pivotal role in controlling gene expression by structuring the bacterial genome. Here, we identified two new Ros/MucR family members in *Sinorhizobium meliloti*. They differ from classical MucR homologs since MucR2 lacks the circular oligomeric structure typical of other family members and MucR3 shows a concentration-dependent oligomerization ability with a low propensity to form circular particles, as shown by cryogenic electron microscopy. Moreover, MucR2 and MucR3 present a new zinc coordination sphere. The newly identified MucRs bind DNA, but lack the DNA bridging activity, which is crucial for structuring the bacterial genome. Using mass spectrometry, light scattering, NMR, EMSA and bridging assay, our study reports the first identification and characterization of two new MucRs and indicates that Ros/MucR family members control gene expression through distinct mechanisms. These results provide an important framework for future studies aimed at dissecting the interplay among MucR proteins and understanding how they can jointly orchestrate condition-dependent gene expression in bacterial species expressing multiple *mucR* homologous genes.

## 1. Introduction

The Ros/MucR protein family includes numerous transcriptional regulators that play a key role in controlling the expression of a wide number of genes, including those required for successful infection of eukaryotic hosts [1,2,3,4,5,6,7,8,9,10,11,12,13,14,15,16,17]. Members of this family are mainly found in α-proteobacteria, a class encompassing both pathogenic species, such as *Brucella* spp., and symbiotic ones like *Rhizobium* spp [18].

The well-studied family members Ros from *Agrobacterium tumefaciens* and MucR from *Brucella abortus* have a DNA binding domain (DBD) folded around a zinc ion, but other members, such as Ml4 and Ml5 from *Mesorhizobium loti*, preserve a functional fold of the DBD in the absence of any metal [19,20].

More recently, several members of the Ros/MucR family have been shown to oligomerize via the oligomerization domain at the N-terminus of the proteins (NTD) [21,22,23,24,25]. The NTD is composed mostly of hydrophobic residues arranged in two α-helices, which mediate monomer–monomer interactions and give rise to a peculiar circular quaternary structure shaped like a truncated cone. In each monomer, the NTD is connected to the DBD through a flexible linker that, in the oligomeric structure, protrudes from the wider rim of the cone [23]. This oligomeric circular assembly enables Ros/MucR proteins to bridge double-stranded DNA segments, forming DNA–protein–DNA complexes, thereby allowing these proteins to organize and structure the bacterial genome [23,24,25]. The ability to form high-order oligomers through an N-terminal oligomerization domain [21,22,23,25], to bind AT-rich DNA by a C-terminal domain [26,27,28,29] and to structure the bacterial genome [23,24,25] has led to the classification of Ros/MucR proteins as a subfamily of H-NS (Histone Nucleoid Structuring)-like proteins [23,24,26]. H-NS-like proteins are well known for structuring and organizing the genome in several bacterial species [30,31,32,33,34,35,36] by binding DNA through a C-terminal DNA-binding domain and oligomerizing via an N-terminal domain [32,36], thus sharing these typical features with Ros/MucR proteins.

In this study, two new members of the Ros/MucR family, named MucR2 and MucR3, have been identified by tandem mass spectrometry (MS/MS) analysis in *Sinorhizobium meliloti*, the α-proteobacterium hosting the first recognized member of this family, called MucR because of the mucoid appearance of the colonies formed by the strain lacking the encoding gene [2,12,24].

Light scattering and cryo-EM analyses reported here show that MucR2 is unable to form oligomeric structure and that MucR3 oligomerizes in a concentration-dependent manner using a hydrophobic residue outside the oligomerization domain identified in the classical MucR proteins [22,23,25]. Moreover, the two newly identified MucRs lack the ability to bridge or structure DNA even if they can bind the same DNA target recognized by the first identified MucR, now called MucR1. Finally, the two new MucR proteins show a new zinc coordination sphere, which may involve a tyrosine residue as a fourth metal coordinating residue that has never been observed in zinc finger domains.

Overall, our findings demonstrate that the Ros/MucR protein family is more heterogeneous in terms of structure and function than expected. Although sharing a high degree of primary sequence identity, some members lack the prototypical quaternary structure, which reflects distinct functions in genome structuring and thus in regulating gene expression. Indeed, MucR2 and MucR3 are not typical H-NS-like proteins, highlighting the existence of a distinct Ros/MucR subgroup that regulates transcription without the ability to structure DNA. This suggests a more complex system of gene expression control mediated by MucR proteins in *S. meliloti* and possibly in many other α-proteobacteria that contain multiple copies of the *mucR* gene [37,38].

## 2. Materials and Methods

### 2.1. Cloning, Protein Expression and Purification

The DNA coding sequences for MucR2 (UniProtKB, protein accession number: O33682, gene name SMa1705) and MucR3 (UniProtKB, protein accession number: Q92ZQ1, gene name SMa0748) were amplified by polymerase chain reaction (PCR) using *S. meliloti* 1021 genomic DNA, extracted with the ULTRAprep Genomic DNA kit (miniprep genomic DNA BAC–Topline), as a template. Primers used in these PCRs are reported in Table S1. The PCR products were purified from agarose gel using a gel extraction kit (Qiagen). Then, the purified DNA fragments and the pET-22b(+) vector were digested with NdeI/BamHI restriction enzymes (New England Biolabs, Ipswich, MA, USA). After digestion, both fragments and the linearized vector were purified from agarose gel and subsequently used for a ligation reaction to obtain mucR2–pET-22b(+) and mucR3–pET-22b(+). The obtained clones were sequenced using the Sanger method.

The deletion of the *mucR2* gene encoding for the C-terminal DBD (spanning from residue 80 to residue 163) was cloned in pET-22b(+) following the same protocol as described above by using mucR2–pET-22b(+) as a template and the couple of primers indicated in Table S1.

Site-directed mutagenesis using PCR was performed to generate the sequences encoding MucR2-T64V, MucR2_27-163_, MucR2_27-163_-T64V, and MucR3-L9R. Primers to introduce the required mutations were designed (Table S1) and employed for PCR using mucR2–pET-22b(+) or mucR3–pET-22b(+) as a template. Following the same procedure as described above, the PCR fragments were gel-purified, double-digested with NdeI/BamHI restriction enzymes, purified again from agarose gel after digestion, and finally ligated into pET-22b(+) to obtain mucR2-T64V–pET-22b(+), mucR2_27-163_–pET-22b(+), and MucR2_27-163_-T64V–pET-22b(+).

mucR1–pET-22b(+) (plasmid construction already reported in Slapakova et al., 2023 [39]), mucR2–pET-22b(+), mucR3–pET-22b(+), mucR2-T64V–pET-22b(+), mucR2_27-163_–pET-22b(+), and MucR2_27-163_-T64V–pET-22b(+) were used to transform *E. coli* Bl21 (DE3) or Rosetta (DE3). A collection of transformed colonies was inoculated in LB medium and grown at 37 °C while shaking (200 rpm). The protein expression was induced with 1 mM IPTG when cultures reached 0.45 OD_600nm_ and was carried out at 28 °C. The growth of cultures and protein expression were stopped when cultures reached 0.75 OD_600nm_ (MucR1 and MucR3 in *E. coli* BL21 (DE3)) or 1.00 OD_600nm_ (MucR2 and its mutated version in *E. coli* Rosetta (DE3)). Bacterial cells were harvested by centrifugation.

^15^N-labelled MucR2_80-163_ was expressed using mucR2_80-163_–pET-22b(+) to transform BL21 (DE3), which was inoculated in minimal medium containing 0.5 g/L ^15^NH_4_Cl as the only nitrogen source and grown at 37 °C while shaking (200 rpm). Expression was induced by adding 1 mM IPTG into the culture. The growth and the expression were stopped when the OD_600nm_ value was 1.00. Bacterial cells were harvested by centrifugation.

Purification of the expressed proteins was carried out as follows: bacterial pellets were resuspended in Tris 25 mM pH 7 (MucR1, MucR3, MucR2-T64V, MucR2_27-163_, MucR2_27-163_-T64V, MucR3-L9R) or Tris 25 mM pH 6.5 (MucR2) or Na_2_HPO_4_ 25 mM pH 6.8 (MucR2_80-163_) and lysed by sonication on ice. Lysate samples were centrifuged for 30 min at 27,400 RCF. The soluble fraction was used to carry out cation exchange chromatography using a HiTrap TM SP HP column (Cytiva), followed by size exclusion chromatography (SEC) performed using a Superdex 200 10/300 column (Cytiva) (for MucR1, MucR3, MucR2-T64V, MucR2_27-163_, MucR2_27-163_-T64V and MucR3-L9) or Superdex 75 10/300 column (Cytiva) (for MucR2 and MucR2_80-163_). For MucR2 mutated variants shown to remain monomeric in solution, SEC was also carried out by using a Superdex S75 column (Cytiva) to compare the elution volume to that of wild-type MucR2.

### 2.2. High-Resolution NanoLC-Tandem Mass Spectrometry (MS/MS)

Aliquots of protein samples (50 µg) were reduced with 10 mM dithiothreitol (DTT, 1 h at 55 °C) and alkylated with 7.5 mM iodoacetamide (IAA, 30 min, in the dark, at room temperature). Enzymatic hydrolyses were performed on reduced and alkylated samples by adding TPCK-treated trypsin with an enzyme/substrate (E/S) ratio of 1:200 (*w*/*w*) for 3 h, 1:100 for 16 h, and 1:50 for 4 h at 37 °C. Mass spectrometry analyses of tryptic samples (200 fmol) were performed on a Q-Exactive Orbitrap mass spectrometer equipped with an EASY-Spray nano-electrospray ion source (Thermo Fisher Scientific, Bremen, Germany) and coupled with a Thermo Scientific Dionex UltiMate 3000 RSLCnano system (Thermo Fisher Scientific). Solvent composition was 0.1% formic acid in water (solvent A) or 0.1% formic acid in acetonitrile (solvent B). Peptides were loaded on a trapping PepMap™100 µCartridge Column C18 (300 µm × 0.5 cm, 5 µm, 100 Å) and desalted with solvent A for 3 min at a flow rate of 10 µL/min. After trapping, eluted peptides were separated on an EASY-Spray analytical column (50 cm × 75 µm ID PepMap RSLC C18, 3 µm, 100 Å) and heated to 35 °C at a flow rate of 300 nL/min using the following gradient: 4% B for 3 min, from 4% to 55% B for 60 min, from 55% to 70% B for 10 min, and from 70% to 95% B for 2 min. Eluting peptides were analyzed on the Q-Exactive mass spectrometer operating in positive polarity mode with capillary temperature of 280 °C and a potential of 1.9 kV applied to the capillary probe. Full MS survey scan resolution was set to 70,000 with an automatic gain control (AGC) target value of 3 × 10^6^ for a scan range of 375–1500 *m*/*z* and maximum ion injection time (IT) of 100 ms. The mass (*m*/*z*) 445.12003 was used as lock mass. A data-dependent top 5 method was applied, during which higher-energy collisional dissociation (HCD) spectra were obtained at 17,500 MS2 resolution with AGC target of 1 × 10^5^ for a scan range of 200–2000 *m*/*z*, maximum IT of 55 ms, 2 *m*/*z* isolation width, and normalized collisional energy (NCE) of 27. Precursor ions targeted for HCD were dynamically excluded for 15 s. Full scans and Orbitrap MS/MS scans were acquired in profile mode, whereas ion trap mass spectra were acquired in centroid mode. Charge state recognition was enabled by excluding unassigned charge states. The acquired raw files were analyzed with Proteome Discoverer 2.4 software (Thermo Fisher Scientific, Rockford, IL, USA) using the SEQUEST HT search engine. The HCD MS/MS spectra were searched against the whole UniProt_SwissProt KB database (version: 2024_05; number of entries: 572,214 sequences) supplemented with MucR3 from *S. meliloti* (AC: Q92ZQ1), assuming trypsin (Full) as digestion enzyme with two allowed numbers of missed cleavage sites. The mass tolerances were set to 10 ppm and 0.02 Da for precursor and fragment ions, respectively. Oxidation of methionine (+15.995 Da) was set as dynamic modification, and carbamidomethylation of cysteine (+57.021 Da) as static modification. False discovery rates (FDRs) for peptide spectral matches (PSMs) were calculated and filtered using the Target Decoy PSM Validator Node in Proteome Discoverer. The Target Decoy PSM Validator Node specifies the PSM confidences based on dynamic score-based thresholds. It calculates the node-dependent score thresholds needed to determine the FDRs, which are given as input parameters of the node. Target Decoy PSM Validator was run with the following settings: maximum Delta Cn of 0.05, a strict target FDR of 0.01, a relaxed target FDR of 0.05 and validation based on q-value. The Protein FDR Validator Node in Proteome Discoverer was used to classify protein identifications based on q-value. Proteins with a q-value of <0.01 were classified as high-confidence identifications, and proteins with a q-value of 0.01–0.05 were classified as medium-confidence identifications. Only proteins identified with medium or high confidence were retained, resulting in an overall FDR of 5%.

### 2.3. Circular Dichroism (CD)

CD experiments were performed using a Jasco J-1500 spectropolarimeter equipped with a Peltier temperature control system at 20 °C. Spectra were collected in the far-UV region, 200–260 nm wavelength range, using a quartz cuvette with a 0.1 cm path length, 20 nm min^−1^ scan speed, 1.0 nm band width, D.I.T. of 4 s, and 0.1 nm data pitch.

All CD samples contained approximately 15 µM of protein in a buffer of 10 mM Tris pH 7.0 supplemented with correct NaCl concentration used to purify the proteins. All spectra were acquired in triplicate, and the background signal of buffer alone was subtracted.

### 2.4. Static Light Scattering (LS)

To measure the molecular weight of MucR1, MucR2, MucR3, MucR2-T64V, MucR2_27-163_, MucR2_27-163_-T64V, and MucR3-L9R, a MiniDAWN Treos spectrometer (Wyatt Instrument Technology Corp, Santa Barbara, CA, USA) equipped with a laser operating at 658 nm was used, connected in-line to an SEC system.

Samples at a concentration of 0.5 mg/mL or 1 mg/mL were loaded in a Superdex 200 10/300 column (GE Healthcare, Mörfelden-Walldorf, Germany) equilibrated in the same buffer as eluted from cation exchange chromatography (25 mM Tris pH 7.0, 0.6 M NaCl for MucR1, MucR2_27-163_-T64V; 25 mM Tris pH 7.0, 0.3 M NaCl for MucR2-T64V, MucR2_27-163_; 25 mM Tris pH 6.5, 0.3 M NaCl for MucR2; 25 mM Tris pH 7.0, 0.5 M NaCl for MucR3 and MucR3-L9R). The column was connected to a triple-angle LS detector equipped with a Quasi-Elastic Light Scattering (QELS) module. A constant flow rate of 0.5 mL/min was applied. Elution profiles were detected by a Shodex interferometric refractometer and a MiniDAWN TREOS LS system. Data were analyzed using Astra 5.3.4.14 software (Wyatt Technology, Santa Barbara, CA, USA).

Triplicates of each experiment were carried out.

### 2.5. Dynamic Light Scattering (DLS) Analyses

The DLS measurements were carried out using a Malvern nanozetasizer (Malvern, Worcestershire, UK). The samples were placed in a disposable cuvette and held at 25 °C. The MucR1, MucR2, MucR3, MucR227-163, MucR2-T64V, and MucR227-163-T64V proteins were assayed at a concentration over the range 14–30 µM. For each sample, we used Tris as dispersant, a refractive index of 1.33, and a viscosity of 0.8872 (mPa.s). The analyses were recorded three times with 11 sub-runs using the multimodal mode. The Z-average diameter was calculated from the correlation function using the Malvern technology software ZS Xplorer version 3.2.0.84.

### 2.6. Cryogenic Electron Microscopy (Cryo-EM) Sample Preparation and Imaging

MucR1, MucR2 and MucR3 at a concentration of 0.5 and 0.2 mg/mL (for MucR1: 3 μM and 1 μM, respectively, considering the MW of the decamer obtained by LS in Slapakova et al. 2023 [39]; 31 μM and 12 μM, respectively, considering the MW of the monomer; for MucR2: 28 μM and 11 μM, respectively, considering the MW of the monomer; for MucR3: 30 μM and 12 μM, respectively, ,considering the MW of the monomer) in 25 mM Tris pH 7.0, 0.4 M NaCl buffer were used for cryo-EM imaging.

Sample vitrification was carried out with a Mark IV Vitrobot (Thermo Fisher Scientific, Bremen, Germany). Three microliters of each protein sample was applied to a Quantifoil R1.2/1.3 Cu 300-mesh grid previously glow-discharged at 30 mA for 30 s in a GloQube (Quorum Technologies, Laughton, East Sussex, UK). The sample was incubated on the grid for 60 s at 4 °C and 100% humidity, blotted, and plunge-frozen into liquid ethane.

Vitrified grids were transferred to a Talos Arctica (Thermo Fisher Scientific) operated at 200 kV and equipped with a Falcon 3 direct electron detector (Thermo Fisher Scientific). MucR1/2/3 were imaged at 120,000×, which corresponds to a pixel size of 0.86 Å/pixel.

### 2.7. Nuclear Magnetic Resonance Spectroscopy (NMR)

The NMR experiments were conducted at 298 K on a Bruker Avance III HD 600 MHz spectrometer equipped with a triple-resonance Prodigy N2 cryoprobe with a *z*-axis pulsed field gradient.

1H-15N HSQC and long-range 2JNH 1H-15N HSQC spectra were acquired as previously described [19]. NMR samples contained 200 µM ^15^N-labeled MucR280-163 in 20 mM phosphate buffer (90:10 H2O: H2O ratio), 0.3 M NaCl, pH 6.8.

All NMR data were processed using Bruker TopSpin 4.0.8 software, and spectra were analyzed using CARA 1.8.3 (downloaded from cara.nmr.ch) and SPARKY 3.12 [40].

### 2.8. AlphaFold3 Prediction

MucR2_80-163_ structural models were generated by means of AlphaFold 3 server. The amino acid primary sequence of the DBD of MucR2 protein (from Ala80 to Lys163) was used as input, as well as a zinc ion. The consistency of the five generated models was evaluated, and the best ranked one (pTM = 0.69 and plDDT higher than 90 for the region Pro79-Tyr137, corresponding to the zinc-binding globular domain) was chosen for further analysis. Models were visualized using ChimeraX software [41].

### 2.9. Electrophoretic Mobility Shift Assay (EMSA)

EMSA experiments were carried out as previously reported [21]. In short, 10 pmol of rem site3 (30 bp) and ndvA (85 bp) double-stranded oligonucleotide was used as a target for protein binding. The two targets were already described in Slapakova et al. 2023 [39] as recognized by MucR1. Their sequences are also reported in Table S1.

Varying amounts of proteins were used as indicated in the text of Section 3. Proteins and DNA targets were mixed in a buffer containing 25 mM HEPES (pH 7.9), 50 mM KCl, 6.25 mM MgCl_2_, and 5% glycerol. Samples were incubated for 10 min on ice and loaded onto a 5% polyacrylamide gel.

Electrophoresis of samples loaded onto 5% polyacrylamide gel was performed in 0.5× TBE at room temperature for 70 min at 200 V. Gels were stained for 20 min using Diamond™ Nucleic Acid Dye (Promega, Madison, WI, USA) following the manufacturer’s instructions and imaged using a Typhoon Trio+ scanner (GE Healthcare).

The results of EMSAs shown in this study are representative of 3 replicates.

The percentage of bound and free DNA in EMSAs with rem site3 target were calculated by Image Tool (GE).

### 2.10. Bridging Assay

A MucR target sequence of 85 bp present in the region upstream of the *ndva* gene was cloned into pBTH154 [42] to obtain pBTH154-ndva. The DNA substrates to be used in the bridging assay were generated by PCR using the pBTH154-ndva as a template and the primers reported in Table S1 (primer 10 and 11). Primer 10 was biotinylated or Cy5-labeled at 5′ to obtain the prey DNA or the bait DNA, respectively. The PCR products were gel-purified, and the concentration was determined by using a NanoDrop spectrophotometer.

Next, 150 µL of hydrophilic streptavidin magnetic beads (New England Biolabs, USA) was washed twice with 750 µL PBS and next resuspended in 750 µL Buffer A (20 mM Tris–HCl, pH7.4, 1 mM EDTA, 500 mM NaCl). Then, 15 pmol biotin-labelled DNA was added to the beads, which were then incubated 30 min at room temperature while gently rotating. After incubation, beads were collected using a magnetic rack, the supernatant was discarded, and beads were washed twice with 550 µL Buffer B (20 mM Tris, pH 7.4, 150 mM NaCl, 1 mM dithiothreitol, 5% glycerol (*v*/*v*), 0.05% Tween 20). After washes, beads were resuspended in 550 µL Buffer B, and 15 pmol Cy5-labeled DNA was added to the beads. The final volume of the suspension was adjusted to 750 µL, and then incubated for 15 min at room temperature while gently rotating.

After incubation, the bead suspension was divided into aliquots of 50 µL. Protein was added into the aliquots at the concentration to be tested. Four aliquots of beads were protein-free, to be used as a control.

All samples were incubated for 30 min at room temperature while gently rotating, then beads were collected by using a magnetic rack. The supernatant derived from protein-free samples was transferred into a clean tube and marked as sample A, while the supernatant derived from protein-containing samples was discarded. Sample A served as a reference corresponding to 100% fluorescence. After beads were washed twice with 150 μL PBS, 60 µL elution buffer (Buffer B supplemented with 0.1% SDS and 20 μg/mL biotin) was added to beads of protein-containing samples and protein-free samples, and the samples were boiled for 10 min. Beads were collected by using a magnetic rack, and supernatants marked as sample B were used to measure fluorescence by using GloMax^®^ Discover. Measures of B derived from protein-containing samples represent the DNA recovery, calculated as a percentage using sample A as a reference. Fluorescence measures of B deriving from protein-free samples were subtracted from protein-containing samples as background signal.

## 3. Results

### 3.1. MucR2 and MucR3 in S. meliloti Are Two New Members of the Ros/MucR Protein Family

**Figure 1:**
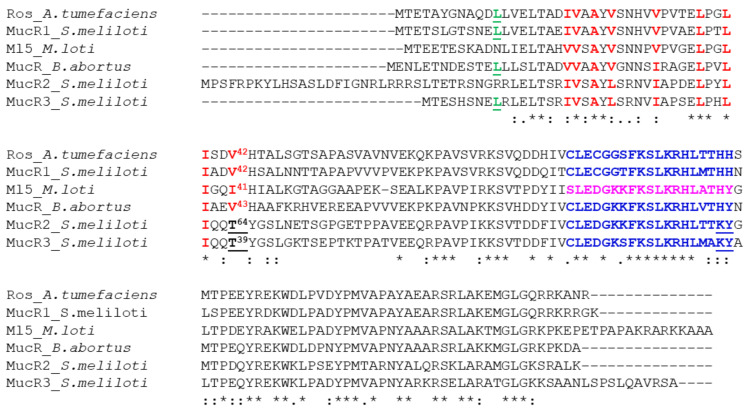
**Sequence alignment of homologous proteins belonging to Ros/MucR family.** In red, conserved residues identified as crucial for oligomerization [22,23,25]. In bold black and underlined, a Thr, present in MucR2 and MucR3, which substitutes a hydrophobic residue of the oligomerization domain of homologous proteins. In blue, the zinc-binding region of the zinc-bound proteins, and in pink, the corresponding region of zinc-free protein Ml5 [19,20,23]. In bold blue and underlined, two residues of MucR2 and MucR3 substituting the zinc coordinating histidines of the zinc-binding region. In green and underlined, the Leu residue present in MucR3 and conserved in *A. tumefaciens* Ros, *S. meliloti* MucR1 and *B. abortus* MucR. Uniprot entry identifiers of the proteins aligned: Ros_*A.tumefaciens*, Q04152; MucR1_*S.meliloti*, P55323; Ml5_*M.loti*, Q98A76; MucR_*B.abortus*, Q2YMR3; MucR2_*S.meliloti*, O33682; MucR3_*S.meliloti*, Q92ZQ1. Asterisks indicate conserved residues, one dot indicates semi-conservative substitutions, two dots indicate conservative substitutions.

The *S. meliloti* MucR protein (hereafter MucR1), homologous to those characterized in *Brucella abortus, Agrobacterium tumefaciens*, and *Mesorhizobium loti*, exhibits the typical sequence signatures of the Ros/MucR family. In particular, the residues essential for oligomerization are conserved as well as the zinc-binding site, which corresponds to the canonical Cys_2_His_2_ motif found in *A. tumefaciens* Ros protein. This contrasts with the Cys–Asp–His variant observed in *B. abortus* MucR [23] and in Ml1, Ml2, and Ml3 from *M. loti* [19,20] (Figure 1).

Jiao and colleagues reported that multiple *mucR* genes can be present in the same bacterial species [37,38]. Analyzing gene data banks in search of MucR homologs, we focused on two *mucR* homologous genes present in *S. meliloti*. We then analyzed the sequences of the putative proteins, hereafter referred to as MucR2 and MucR3, encoded by these two genes. MucR2 and MucR3 share 50–80% sequence identity despite some notable differences (Figure 1).

Most residues critical for oligomerization are conserved in MucR2 and MucR3, except for Thr64 in MucR2 and Thr39 in MucR3, which replace the Val or Ile residues typically found at the corresponding position in Ros/MucR proteins (Figure 1) [25]. The zinc-binding region of MucR2 and MucR3 is also mostly conserved (Figure 1). In this region, the first three residues coordinating zinc, Cys-Asp-His, previously identified in *B. abortus* MucR [23] and present in many other homologs [19], are conserved. Interestingly, in MucR2 and MucR3, the histidines that serve as the fourth zinc-coordinating position in Ros [43,44,45,46,47] are absent and are replaced by lysine or tyrosine (Figure 1). Additionally, MucR2 features a prolonged N-terminal region of about 20 residues, unprecedented among known Ros/MucR proteins (Figure 1).

To investigate whether the two newly identified members of the Ros/MucR family, MucR2 and MucR3, were expressed in *S. meliloti*, we performed ion exchange chromatography on total soluble protein extract from *S. meliloti* 1021 cultured until reaching the stationary phase. Based on theoretical isoelectric points calculated by the ProtParam tool (https://web.expasy.org/protparam/ (accessed on 1 May 2026)) (pI 10.11 for MucR2 and pI 9.8 for MucR3), we employed cation-exchange chromatography at pH 7, under which MucR proteins were expected to be positively charged. The eluted fractions were analyzed by high-resolution nanoLC-MS/MS (Figure S1a). A data-dependent acquisition mode was used, during which higher-energy collisional dissociation (HCD) MS/MS spectra were obtained for the five most intense mass peaks in each scan, allowing for accurate amino acid sequencing of tryptic peptides. Amino acid sequences of peptides obtained by high-resolution tandem mass spectrometry are reported in Table 1. Representative MS/MS spectra of ions mapped on MucR1, MucR2 and MucR3 are reported in Figure S1b–d, respectively.

Overall, a coverage of about 41% and 51% of the protein sequences was obtained for MucR1 (59 out of 143 amino acid residues) and MucR2 (83 out of 163 amino acid residues), respectively. A higher 79% coverage was obtained for MucR3 (118 out of 149 residues). These results show that the two Open Reading Frames, SMa1705 and SMa0748, encode two genes transcribed and translated in *S. meliloti*, thus identifying MucR2 and MucR3 as part of the *S. meliloti* proteome.

### 3.2. MucR2 Is a Monomer in Solution, Whereas MucR3 Oligomerizes at High Concentration

Classical Ros/MucR proteins form higher-order oligomers with a characteristic circular structure that allows them to bridge DNA [23,25].

**Figure 2:**
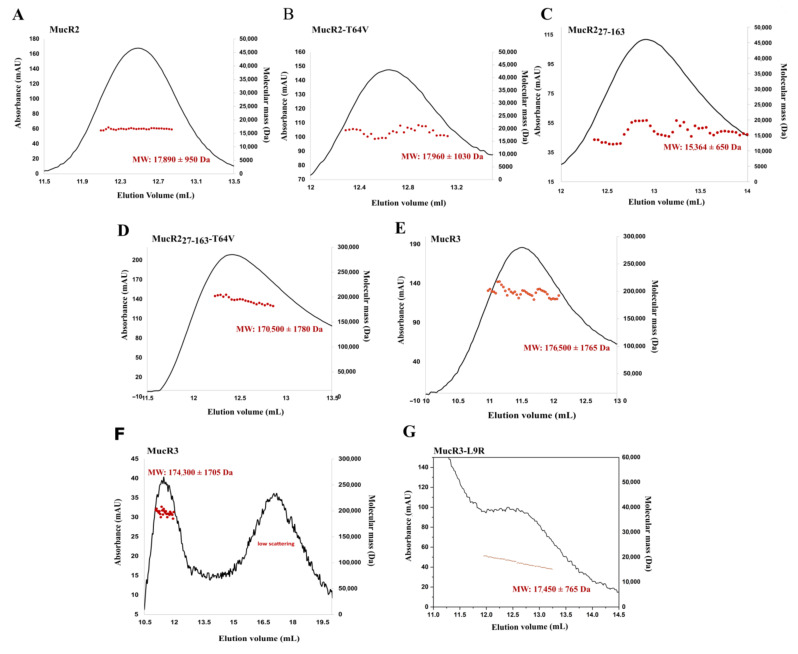
**LS of MucR2, MucR3 and their mutants.** (**A**,**D**–**G**) show the results of LS carried out at a protein concentration of 30 µM. The same results were obtained when 60 µM of proteins was tested; (**B**) represents LS of MucR3 carried out at 30 µM; (**C**) represents LS of MucR3 carried out at 60 µM. The MW of each protein with the relative error is indicated.

Recombinant MucR2 and MucR3 were purified to investigate their ability to oligomerize. The proper fold of the purified proteins was verified by circular dichroism (CD) (Figure S2). Light scattering (LS) methods were used to determine the molecular mass and the oligomerization states of the proteins (Figure 2A–C).

LS analysis showed that MucR2 has a molecular weight (MW) consistent with a monomer in solution at either 30 μM or 60 μM (Figure 2A), whereas MucR3 displayed a different behavior at the two concentrations tested (Figure 2B,C).

LS analysis of 30 µM MucR3 samples displayed a molecular weight (MW) compatible with a decameric assembly (Figure 2B). Interestingly, analysis of UV absorption peaks during LS revealed a second fraction of MucR3 that failed to produce a clear scattering signal, but eluted at a volume corresponding to a monomer (Figure 2B). This latter peak disappeared when MucR3 concentration reached 60 µM (Figure 2C), suggesting that the oligomerization state of this protein is concentration-dependent.

**Figure 3:**
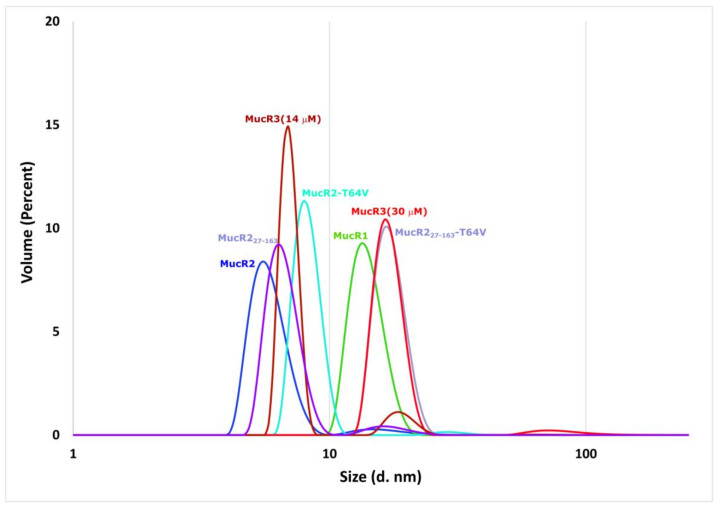
**DLS of MucR2, MucR2 mutated versions and MucR3**. MucR1, MucR2 and MucR2 mutated versions do not show changes in measured diameter at the different concentrations tested. In the figure, only one point for each of these proteins is reported. For MucR3, whose measured diameter changes over a concentration range of 14–30 μM, the measurements at the lowest (14 μM) and the highest (30 μM) concentration are shown in the figure.

To further analyze the ability of MucR2 and MucR3 to oligomerize, dynamic light scattering (DLS) was performed over a concentration range of 14–30 μM. As a reference for the MucR oligomeric radius calculation, the 14 μM MucR1 sample, already reported to be oligomeric [39], was first analyzed (Figure 3).

MucR2 showed a diameter consistent with a monomeric state even at 30 µM concentration (Figure 3). In contrast, at 14 µM, MucR3 exhibited a diameter corresponding to a monomer, but as the concentration increased to 30 µM, the measure of the diameter reached a value comparable to that observed for the oligomeric MucR1 (Figure 3).

To investigate the structural elements impairing the ability of MucR2 to oligomerize, we designed three MucR2 variants: (i) MucR2_27-163_, in which the prolonged N-terminus of MucR2 was deleted; (ii) MucR2-T64V, in which the Thr in position 64 of MucR2 was replaced by a Val, present in MucR1 and proved to be involved in the oligomerization [25]; and (iii) MucR2_27-163_T64V, bearing both the deletion of the prolonged N-terminus and the T64V point mutation. CD spectra of the expressed and purified MucR2 mutants showed that the proteins were folded (Figure S2). The three MucR2 mutants were analyzed by LS and DLS. The results showed that MucR2_27-163_ and MucR2-T64V are monomeric (Figure 2D,E and Figure 3). In contrast, the MucR2_27-163_T64V protein gains the ability to oligomerize (Figure 2D and Figure 3). These results demonstrate that both the prolonged N-terminus and the lack of the valine in position 64 are sufficient to prevent MucR2 oligomerization; the absence of prolonged N-terminus together with the presence of the valine residue are necessary to restore the oligomerization ability.

To explore the structural elements responsible for concentration-dependent oligomerization of MucR3, we compared its primary sequence to that of MucR2 and other Ros/MucR proteins. MucR3 presents a Leu residue in position 9 that is absent in MucR2 but conserved across Ros and MucR proteins (Figure 1). Based on the hydrophobic nature of the interactions allowing the oligomerization of MucR proteins [23,25], we designed a MucR3 mutant version, named MucR3-L9R, in which Leu9 was substituted by an Arg, as it is present in the monomeric MucR2. CD spectra of MucR3-L9R showed that the protein was folded (Figure S2). LS analysis demonstrated that this MucR3 mutant version is monomeric in solution even at 60 μM (Figure 2G), indicating that Leu9 contributes to concentration-dependent oligomerization by triggering and stabilizing hydrophobic interactions within the oligomerization domain as the protein concentration increases.

**Figure 4:**
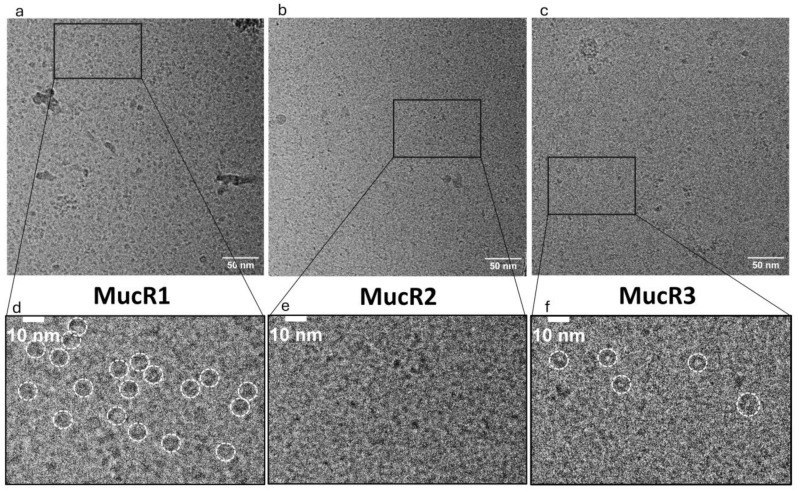
**High-magnification micrographs of MucR1, MucR2 and MucR3 proteins vitrified at 0.5 mg/mL**. (**a**–**c**) represent MucR1, MucR2 and MucR 3 images, respectively, at 120,000× magnification. (**d**–**f**) display close-ups of MucR1, MucR2 and MucR3 where circular oligomeric particles have been highlighted, when present, by a white dotted frame. The experiment confirmed evidence obtained in LS and DLS experiments: a marked propensity of MucR1 to oligomerize in circular cone-shaped particles; a propensity of MucR3 to oligomerize, despite a lower number of single particles being detected; MucR2 oligomeric particles could not be detected.

To further investigate the different oligomerization abilities of MucR1, MucR2, and MucR3, and to qualitatively evaluate the quaternary structure of their oligomers, cryo-electron microscopy (cryo-EM) analysis was performed. As expected, samples of MucR1 confirmed the typical NTD circular assembly previously reported for MucR family oligomers [23] (Figure 4). Using the same protein concentration applied for MucR1, we also collected cryo-EM images of MucR2 and MucR3. No oligomeric particles were detected for MucR2, while MucR3 displayed only a few circular oligomeric particles compared to MucR1, confirming a lower propensity to form oligomers than typical MucR proteins (Figure 4).

### 3.3. MucR2 and MucR3 Bind Zinc with a Peculiar Coordination Sphere

**Figure 5:**
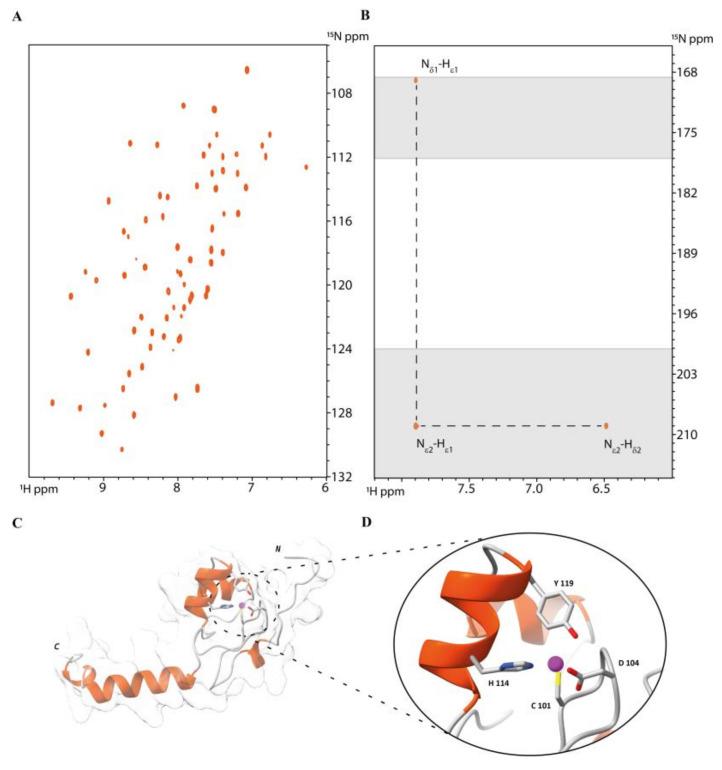
**NMR conformational analysis and AlphaFold3 structural prediction of MucR2 C- terminal DBD.** (**A**) 1H-15N HSQC spectrum of MucR2_80-163_. (**B**) Portion of the long-range 2JNH 1H-15N HSQC spectrum of MucR2_80-163_. The zinc-binding histidine chemical shift ranges are highlighted in grey. The His 114 cross-peaks connected by the dotted line present the classical pattern of a zinc-binding histidine via Nε2 nitrogen. (**C**,**D**) Ribbon drawing representation of the best-score MucR2_80-163_ structural model as revealed by AlphaFold3. Side chains of the coordinating residues are illustrated as light-grey sticks; the zinc ion is reported in magenta.

To investigate the ability of these two new Ros/MucR proteins to bind zinc even with their peculiar metal coordination sphere (Figure 1), the MucR2 C-terminal domain, MucR2_80-163_, corresponding to the DNA-binding domain of *A. tumefaciens* Ros (Ros_56-142_ [43,44,45,46,47]) and *B. abortus* MucR (MucR_57-142_ [23]) was studied as a model using NMR. The 1H-15N HSQC spectrum of MucR2_80-163_ (Figure 5A) shows a quite good signal distribution along both dimensions (3 PPM for the proton and 25 PPM for the nitrogen), indicating that the protein reorganizes into secondary and tertiary structures in solution. Due to the presence of a single histidine residue in the whole sequence of the protein, the His 114 tautomeric form was investigated via the acquisition of a long-range 2JNH 1H-15N HSQC (Figure 5B). This experiment, indeed, allows the characterization of the protonation state of the nitrogens of the imidazole ring of this amino acid and discrimination of its involvement in zinc binding [19]. The cross-peak pattern clearly shows that His114 is Nδ1-H tautomer (i.e., with the Nε2 de-protonated), which is the typical tautomeric form assumed by histidine imidazole rings in the prokaryotic zinc-bound domains [19]. Furthermore, both Nε2 and Nδ1 chemical shifts (209.10 and 169.05 ppm, respectively) are in the typical chemical shift range found for zinc-binding histidine side chains [19]. These results demonstrate that the MucR2 C-terminal region spanning from the Ala in position 80 to the Arg in position 163 can bind zinc, which is coordinated by His114 in the third position of the zinc coordination sphere. Based on sequence alignment (Figure 1), this latter likely includes Cys101 and Asp104, thus reproposing the previously studied zinc-bound *B. abortus* MucR in the first three zinc coordinating residues [23]. To further investigate the zinc coordination sphere and to obtain additional structural details, we performed an AlphaFold3 prediction of the MucR2_80-163_ structure. Indeed, AlphaFold3 implemented previous version of AlphaFold by providing the possibility to add several metal ions, including Zn^2+^, forecasting the residues involved in the coordination. As reported in Figure 5C,D, the obtained structural model shows the same globular fold characteristic of the other Ros/MucR homologs (Figure 5C) [23,39,43]. The AlphaFold3 model indicates that the first three Zn^2+^ coordinating positions are Cys, Asp, and His and suggests that the fourth one might be occupied by Tyr 119, which could bind the metal with the oxygen of the phenolic group, with the Lys 118 side chain protecting the metal ion center from the water bulk (Figure 5C).

On the basis of the predicted MucR2_80-163_ structure, we designed a double mutant version of MucR2_80-163_, in which the Lys 118 and the Tyr 119 are replaced by Ala (MucR2_80-163_K118AY119A).

The 1H-15N HSQC spectrum acquired for this double mutant is typical of a largely unfolded polypeptide, thus demonstrating that both Lys118 and Tyr119 play a crucial role in the MucR2_80-163_ fold. The two residues might play a role in stabilizing the metal ion coordination sphere and the whole protein structure with it.

Further analyses will be required to assess the affinity for zinc and unequivocally identify the fourth residue coordinating the metal.

### 3.4. MucR2 and MucR3 Bind DNA, but Do Not Show Bridging Activity

**Figure 6:**
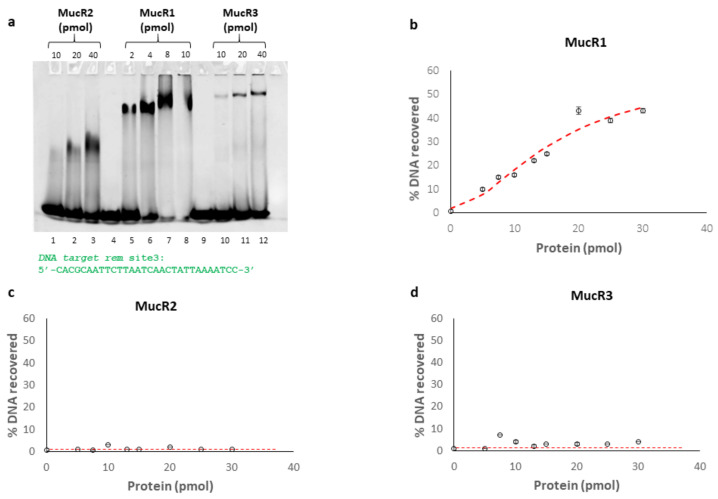
**Binding and bridging experiments with MucR2 and MucR3.** (**a**) EMSA with MucR1, MucR2 and MucR3. The 30 bp rem site3 [39] was used as a DNA target. The sequence is indicated in green. Names of the proteins and the amounts used in the reactions are indicated at the top of the lanes. The binding reaction was performed in 20 μL. The concentrations of protein in the reaction were 0.5 μM, 1 μM and 2 μM for MucR2 (lanes 1, 2 and 3, respectively) and MucR3 (lanes 10, 11, 12, respectively); the concentrations of MucR1 in the reaction were 0.1 μM, 0.2 μM, 0.4 μM, and 0.5 μM (lanes 5, 6, 7, 8, respectively). In lanes 4 and 9, only DNA was loaded. (**b**–**d**) Bridging assay with MucR1, MucR2 and MucR3, respectively. The amounts of protein used are indicated on the *X* axis, the percentage of DNA recovered on the *Y* axis.

To test the DNA-binding ability of MucR2 and MucR3, we performed EMSAs with these proteins using the 30 bp double-stranded oligonucleotide (rem site3) as a target (Figure 6a). This DNA sequence is recognized by MucR1 [39], whose DNA-binding domain shares 57% and 63% identity with MucR2 and MucR3, respectively (Figure 1).

Both MucR2 and MucR3 bind the rem site3 target although a higher amount of proteins compared to MucR1 is necessary to form the protein–DNA complex. In fact, MucR1–DNA complex was detectable at a protein/DNA ratio of 1:5, whereas MucR2 and MucR3 required a 1:1 protein/DNA ratio to form a detectable complex. The percentage of the bound and free DNA in EMSAs performed with the three MucR proteins is also reported in Table S2.

The band corresponding to MucR2–DNA shows a faster electrophoretic mobility and a less sharp appearance compared to the MucR1– and MucR3–DNA complexes. This result is expected due to its monomeric structure. Indeed, EMSAs performed with the monomeric mutant version of *B. abortus* MucR [22] showed the same behavior as MucR2 in EMSAs. The less sharp appearance of the protein–DNA complex band could be due to the instability of the complex and/or the possible interaction of MucR2 with different regions of the 30 bp oligonucleotide showing many AT succeeding bases, which are all potential binding sites for MucR proteins [26,27,28]. The MucR3–DNA complex, instead, shows a band with a electrophoretic mobility comparable to that of the MucR1–DNA complex. This could be explained by the ability of MucR3 to oligomerize, although with a low propensity. The oligomeric MucR3 particles could form stable complexes with DNA, thus appearing as a sharp band with a typical electrophoretic mobility of MucR proteins able to oligomerize.

The calculated percentages of bound and free DNA reported in Table S2 indicate that MucR3 binds DNA with a lower affinity compared to MucR1 and MucR2. This could be due to the presence in solution of monomeric and oligomeric protein particles. Given that the electrophoretic mobility of the MucR3–DNA complex is comparable to that of MucR1–DNA, the result of the lower MucR3 affinity might be due to the inability of the monomeric protein fraction to form a stable complex with DNA.

The circular oligomeric structure characteristic of MucR proteins enables DNA bridging, resulting in the formation of DNA–protein–DNA complexes, as was demonstrated for *B. abortus* MucR [23]. We performed bridging assays with MucR1, which shares 92% identity with *B. abortus* MucR, using the ndva sequence (Table S1) as a target, a site known to be recognized by this protein [39]. The results confirm that MucR1 can bridge DNA (Figure 6b).

Then, we carried out bridging assays with MucR2 and MucR3, which can bind the ndva target in EMSAs (Figure S3). As expected, given its monomeric structure, MucR2 cannot bridge DNA (Figure 6c), confirming that this activity requires oligomer formation [23]. Bridging assays with MucR3 show that this protein also fails in forming DNA–protein–DNA complexes (Figure 6d) even when the concentration of the protein used corresponds to that necessary to obtain MucR3 oligomeric structure (see LS and DLS results in this study). This result is consistent with the analyses by cryo-EM (Figure 4), which revealed that MucR3 has a lower propensity than MucR1 to form the circular oligomeric assemblies responsible for bridging activity [23]. Notably, MucR3 carries a substitution of one of the residues (Thr 39 instead of Val) identified as crucial for forming stable MucR oligomers (Figure 1) [25], and its concentration-dependent oligomerization appears to rely mostly on Leu9, a residue located outside the oligomerization domain. The inability of MucR3 to bridge DNA may, therefore, result from the instability of the oligomeric structure and the low propensity of this protein to form circular particles, as shown by cryo-EM experiments. Indeed, the oligomerization of MucR3 by using a residue outside the oligomerization domain might result in an alteration of the typical geometry of the oligomeric particles formed by the classical MucR proteins, which can bridge DNA. Thus, together with the low propensity of MucR3 to form oligomers, differences in the oligomeric structure formed by MucR3 could be a reason for the inability of this protein to bridge DNA even when oligomers are formed.

Finally, we performed the bridging assay with the mutant MucR2_27-163_T64V, which turns out to be able to oligomerize (see LS data in this manuscript). The result (Figure S4) shows that this protein can bridge the ndva target and definitively demonstrates that oligomerization depends on the ability of MucR proteins to form the typical quaternary oligomeric structure through the residues identified in the NTD as crucial for oligomerization. Moreover, this finding further strengthens the conclusion that MucR3 oligomers cannot bridge DNA because of their instability and differences in the quaternary structure.

## 4. Discussion

Ros/MucR proteins have long been described as classical transcriptional regulators [1,2,3,4,5,6,7,8,9,10,11,12,13,14,15,16,17]. However, recent studies have revealed that these proteins can form higher-order oligomers whose structure allows them to bridge and organize DNA [23,25]. This ability to structure DNA has led to the classification of Ros/MucR proteins as a new subfamily of Histone-like Nucleoid Structuring (H-NS) proteins [23,24,25,26].

Our study is focused on two newly identified MucR family members in *S. meliloti*, namely MucR2 and MucR3. Surprisingly, both proteins are unable to form oligomers, unlike all previously characterized members of the Ros/MucR family. This striking absence of quaternary structure for MucR2 and the concentration-dependent oligomerization of MucR3 is mirrored by their functional divergence from the classical MucR protein, now referred to as MucR1.

**Figure 7:**
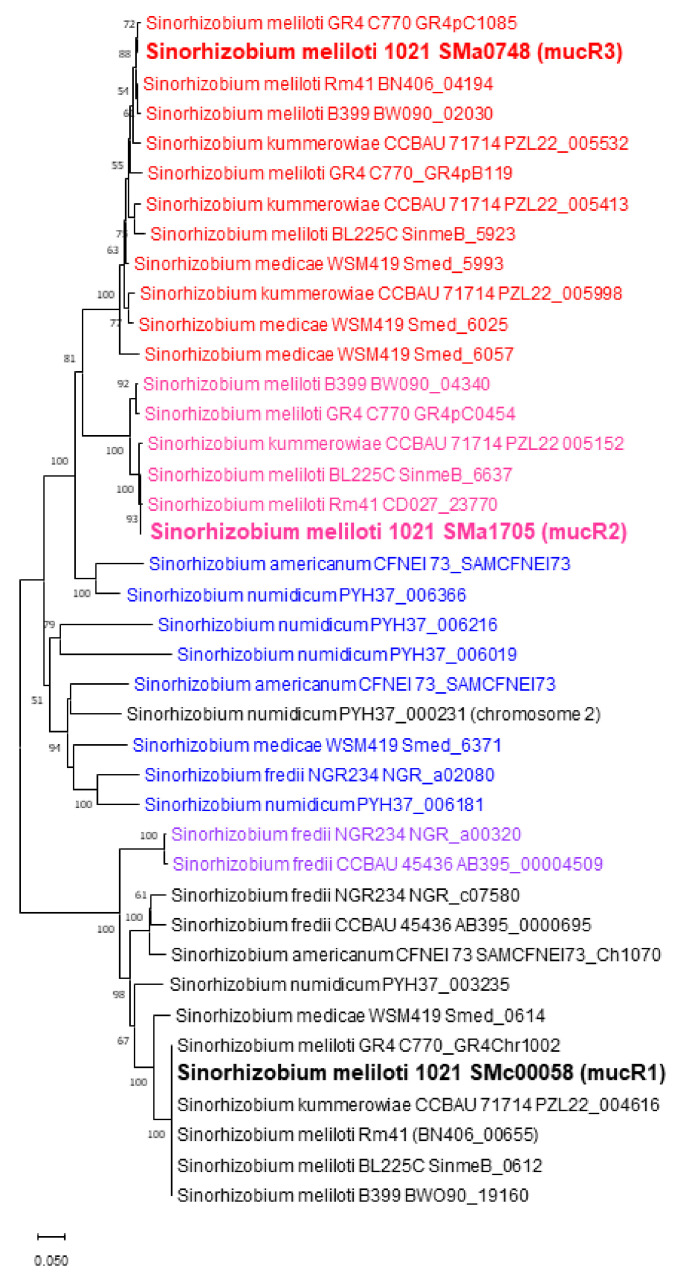
**Neighbor-Joining phylogenetic three of *mucR* genes in *Sinorhizobia*.** Only bootstrap values above 50% are shown. In black letters are genes of chromosomal location; colors are used for genes located in plasmids. Note that, with few exceptions, two well-differentiated gene clusters can be observed: chromosomal versus plasmid genes.

Our results show that MucR2 and MucR3 can bind DNA but lack DNA bridging activity and consequently, they turn out to be unable to structure DNA. This striking finding indicates that a subgroup of MucR homologs has evolved to play a different role from that of the classical MucR proteins. In fact, in addition to the chromosomal *mucR1* gene, all sinorhizobial genomes contain 1–5 additional *mucR* genes that display apparently disparate phylogenies and (with only one exception, *S. numidicum*) are always plasmid-borne, usually a symbiotic plasmid. *mucR2*- and *mucR3*-like genes form two separate but closely related subclusters, and are present only in the species *S. meliloti*, *S. medicae*, and *S. kumerowiae* (Figure 7). The plasmid-borne origin of *mucR2* and *mucR3* genes leads us to hypothesize a potential role of MucR2 and MucR3 proteins in regulating the transcription of the chromosomal *mucR1* gene. The two new MucRs could be acquired and kept during evolution as modulators of *mucR1*, whose correct and fine-tuned expression is beneficial for *S. meliloti* considering the high number of genes controlled by MucR1. Indeed, a fine-tuned expression of the *mucR1* gene would allow differential expression of the encoded protein during the *S. meliloti* cell cycle and in different phases of growth.

Further investigations will uncover the mechanisms by which MucRs regulate gene expression and the interplay among the different MucR proteins, which is unknown so far. Our findings suggest a more complex scenario in the regulation of genes crucial for many aspects of bacterial life.

MucR2 and MucR3 are shown to be DNA-binding proteins that can recognize the target sites of MucR1. The high sequence identity of their DBDs with that of other homologs and the retained ability to bind a zinc ion suggest that the overall fold of their DBD is similar to that already studied in Ros and MucR. Nevertheless, MucR2 and MucR3 display a peculiar zinc coordination sphere formed by a Cys, an Asp and His residues as first, second and third coordinating residues, respectively, while as fourth coordinating residue, an unusual amino acid, such as a Tyr, might substitute the typical His residue. An adjacent Lys residue likely contributes to stabilizing the metal center by shielding the zinc ion from solvent exposure. This peculiar feature warrants a deeper structural investigation not only to verify further the role of the Tyr–Lys couple in zinc coordination, but also to explore potential altered cross-talk between the N-terminal and C-terminal regions. Indeed, given that MucR2 is monomeric and MucR3 is able to form oligomers in a concentration-dependent manner by using a hydrophobic residue (Leu 9) located outside the NTD, the absence of the typical hydrophobic interactions that drive NTD-mediated oligomerization in classical MucRs could alter inter-domain interactions between the NTD and DBD, potentially resulting in subtle structural rearrangements that affect DNA recognition or regulatory function.

Overall, our findings demonstrate that the Ros/MucR family is formed by proteins showing different functional structures that reflect a different way to interact with DNA. Members of this family have been shown to be surprisingly adaptable in their structure and function, while preserving the fundamental capability to interact with DNA even when bridging activity—which depends on the oligomeric structure—is lost. These findings open new perspectives for further studies on a multifaceted protein family whose members can function as H-NS-like proteins [48], counter-silenced by cyclic-di-GMP [49], when able to oligomerize and bridge DNA, or even might act as classical transcription factors by binding DNA [24] when oligomerization ability is missing.

## 5. Conclusions

In this study, two new Ros/MucR proteins have been identified in *S. meliloti.* Through structural and functional experiments, we have demonstrated that the two new Ros/MucR family members, named MucR2 and MucR3, differ from those already known, since MucR2 lacks the oligomeric structure, and MucR3 shows a concentration-dependent oligomerization ability. Moreover, MucR3 can oligomerize by using a hydrophobic residue that is not included in the typical residues responsible for the oligomerization of the classical MucR homologs. As a consequence of these differences in the structure and in the ability to oligomerize, MucR2 and MucR3 cannot bridge DNA. In addition, MucR2 and MucR3 show an unusual zinc coordination sphere, which might be responsible for structural differences in the geometry of the globular fold of their DBDs when compared to the classical Ros/MucR proteins. Thus, the features of the two new MucRs show that the Ros/MucR family is very heterogeneous, being formed by the classical members that can bridge DNA and work as H-NS-like proteins, but also comprising proteins which work mainly as monomers unable to structure DNA by bridging activity even if able to bind DNA.

## Figures and Tables

**Table 1:** Amino acid sequences of tryptic peptides mapped on MucR1 (AC: P55323), MucR2 (AC: O33682) and MucR3 (AC: Q92ZQ1), obtained by high-resolution nanoLC-MS/MS. Sequence, protein accession code (AC), number of missed cleavage sites (MC), charge state, experimental masses of precursor ions, and experimental and theoretical molecular weights of peptides (MH+), together with mass accuracies and retention times, are reported.

Sequence	AC	MC	Charge	*m*/*z* (Da)	Exp Mr [M+H^+^]^+^	Th Mr [M+H^+^]^+^	Δ (ppm)	RT (min)
HLMTHHNLSPEEYR	P55323	0	3	588.613	1763.826	1763.828	0.93	32.4
SVQDDQITCLECGGTFK	P55323	0	2	979.435	1597.862	1597.863	0.02	42.2
DKWDLPADYPMVAPAYAEAR	P55323	1	3	760.367	2279.088	2279.080	3.82	46.3
HLMTHHNLSPEEYR	P55323	0	3	588.614	1763.826	1763.828	-0.93	32.5
NVIAPDELPYLIQQTYGSLNETSGPGEYPPAVEEQRPAVPIKK	O33682	1	4	1203.354	4810.395	4810.386	1.84	47.8
SVTDDFIVCLEDGKK	O33682	1	3	575.950	1725.837	1725.836	0,65	44.2
KSVTDDFIVCLEDGKK	O33682	2	3	618.649	1853.932	1853.931	0.67	37.4
SVTDDFIVCLEDGK	O33682; Q92ZQ1	0	2	799.373	1597.738	1597.741	−1.85	45.4
LPSEYPMTAR	O33682	0	0	582.790	1164.573	1164.572	1.41	32.9
YGMTPDQYR	O33682	0	2	565.751	1130.494	1130.493	0.7	31.8
NYALQR	O33682	0	2	382.705	764.403	764.405	2.57	26.6
MTESHSNELR	Q92ZQ1	0	3	401.852	1203.542	1203.542	0.32	24.5
TSEPTKTPATVEEQRPAVPIKK	Q92ZQ1	2	5	482.269	2407.318	2407.319	0.42	30.7
KSAANLSPSLQAVR	Q92ZQ1	1	3	481.276	1441.812	1441.812	0.12	33.5
IVSAYLSR	Q92ZQ1; O33682	0	2	454.763	908.519	908.520	0.59	35.2
YALTPEQYR	Q92ZQ1	0	2	570.788	1140.568	1140.568	0.42	35.9
LPADYPMVAPNYAR	Q92ZQ1	0	2	789.392	1577.777	1577.778	0.67	40.3
SVTDDFIVCLEDGK	Q92ZQ1; O33682	0	2	799.374	1597.740	1597.741	0.94	46.3
NVIAPSELPHLIQQTYGSLGK	Q92ZQ1	0	3	755.747	2265.229	2265.223	2.49	48.9
ATGLGKKSAANLSPSLQAVRSA	Q92ZQ1	2	2	1027.066	2053.126	2053.140	6.70	48.1

## Data Availability

The original contributions presented in this study are included in the article/Supplementary Materials. Further inquiries can be directed to the corresponding authors.

## Supplementary Materials

The following supporting information can be downloaded at: https://www.mdpi.com/article/10.3390/biom16060781/s1, Table S1: Primers and Oligonucleotides; Table S2: Analysis of EMSAs results; Figure S1: Schematic overview of the experimental workflow used for the peptide mapping of MucR1, MucR2 and MucR3 and MS/MS fragmentation spectrum; Figure S2: CD spectra; Figure S3: EMSA with MucRs and ndva target; Figure S4: Bridging assay with MucR2_27-163_T64V and ndva DNA target.

## Abbreviations

The following abbreviations are used in this manuscript:

CD	Circular Dichroism
Cryo-EM	Cryogenic Electron Microscopy
EMSA	Electrophoretic Mobility Shift Assay
H-NS	Histone-like Nucleoid Structuring
HSQC	Heteronuclear Single Quantum Coherence
LS	Light Scattering
DLS	Dynamic Light Scattering
