# Supplementary material for: Identifying Two New Ros/MucR Proteins: An Atypical Structure with a Divergent Function
Source: Biomolecules. 2026 May 26;16(6):781. doi: 10.3390/biom16060781 (PMC13297338; doi:10.3390/biom16060781)
Supplement: Supplementary file 1 [file biomolecules-16-00781-s001.zip › biomolecules-4294299-supplementary.pdf]

PRIMER	SEQUENCE 5’- 3’
Primer 1: cloning_MucR2_F	5‘ TCG TCT CAT ATG CCT TCG TTC CGA CCG AAA TAT CTG 3‘
Primer 2: cloning_MucR2_R	5’ ACG TCC TCG AGC TAC TTC AAC GCC CGG CTC TTT CCC AGC 3‘
Primer 3: cloning_MucR2Δ27_F	5' CGA CTC CAT ATG ACG GAA ACG CGC TCA AAC 3'
Primer 4: cloning_MucR2_T64V_1	5' TTC GTT CAG GGA GCC GTA GAC TTG CTG GAT AAG ATA 3'
Primer 5: cloning_MucR2_T64V_2	5’ TAT CTT ATC CAG CAA GTC TAC GGC TCC CTG AAC GAA 3’
Primer 6: cloning__MucR3_F	5' ATC TCC ATA TGA CGG AAT CGC ACT CAA ACG AAC TAA GG 3'
Primer 7: cloning__MucR3_R	5'GCG TAG GAT CCT CAG GCC GAT CTT ACC GCT TGC AG 3'
Primer 8: cloning__MucR_L9R_F	5’ TTA CTC ATA TGA CGG AAT CGC ACT CAA ACG AAC TAC TGC TTG AGC TCA CCA GCC GC 3’
Primer 9: cloning__MucR2_80_163_F	5’ TCG TCT CAT ATG GCA GTC GAA GAA CAA CGC CCC GCG GTC CCC 3‘
Primer 10: bridging_Caserta_1	5‘ GCCTCTAGACATGAGCACCGACGTCAGCAGTG 3’
Primer 11: bridging_Caserta_2	5’ GTCCTGGGAGTGCCGGGAGAGCATCAGCATG 3’
Primer 12: S_mel_ΔmucR1_F	5’ GATTCCGATAGGAGAAAGAATCCGGGCGGCCCCAC 3’
Primer 13: S_mel_ΔmucR1_R	5’ GCCCAGTGGGGCCGCCCGGATTCTTTCTCCTATCGGAATCCAGCG 3’
Primer 14: S_mel_ΔmucR1_F_1	5’ATAACAATTTCACACAGGAAACAGCTATGACATGATTACGCGCCCGAGCCAGGCCG 3’
Primer 15: S_mel_ΔmucR1_R_1	5’GATCGGTGCGGGCCTCTTCGCTATTACGCCAGCTGGCGAACCCGAAAGGCGGTTGAAGC3’
Primer 16: S_mel_ΔmucR2_F	5‘ AAG TGC GAT GAT GAC AAA AAG AGC 3‘
Primer 17: S_mel_ΔmucR2_R	5‘CTT CTC GGC ACC GCG AAA AA 3‘
Primer 18: S_mel_ΔmucR2_F_2	5‘ATA GCG ACT CGC GGC TCA TAG TGC GCT AAC CAC TAC TTA CT 3‘
Primer 19: S_mel_ΔmucR2_R_2	5‘ GTAA GTA GTG GTT AGC GCA CTA TGA GCC GCG AGT CGC TAT 3‘
Primer 20: S_mel_ΔmucR3_F	5‘GCC TGT GTT CGA GAA GCC TT 3‘
Primer 21: S_mel_ΔmucR3_R	5‘ACT TCA GGC GGC TCA AGA C 3‘
Primer 22: S_mel_ΔmucR3_F_2	5‘ GTA ACA GCT TGT GGA GAA GAT CAA GTT CGG CTG ATT TCG GCT G 3‘
Primer 23: S_mel_ΔmucR3_R_2	5‘CAG CCG AAA TCA GCC GAA CTT GAT CTT CTC CAC AAG CTG TTA C 3‘
Primer 24: mucR1_RT_F	5‘ACATGTCTCGAATGCGGCGG 3’
Primer 25: mucR1_RT_R	5‘ ACCCATTTCCTTGGCGAGCC 3’
Primer 26: mucR2_RT_F	5‘TACGGCTCCCTGAACGAAAC 3’
Primer 27: mucR2_RT_R	5‘TTGTCGTCAAATGCCGCTTG 3’
Primer 29: mucR3_RT_F	5’ AAAAGTGGAAGCTCCCGGCC 3’
Primer 30: mucR3_RT_R	5‘TTACCGCTTGCAGAGACGGG 3’
Primer 31: rpoD_RT_F	5‘GGACGAGCTGATCAAGGCCG 3’
Primer 32: rpoD_RT_R	5‘CCATAGGATTCGGCCAGGCG 3’
**OLIGONUCLEOTIDE**	**SEQUENCE 5’- 3’**
ndvA	5’GATAATTCTTCATATCACGCCCGGTCTTGCAGGAAACCTGCAAAAAC TCGGAGTCGAGGCATCAAGATAGCTAACGATGAATCAT 3’

Supplementary Table S1: Primer sequences used for PCRs and oligonucleotide sequences tested in bridging assays are reported.

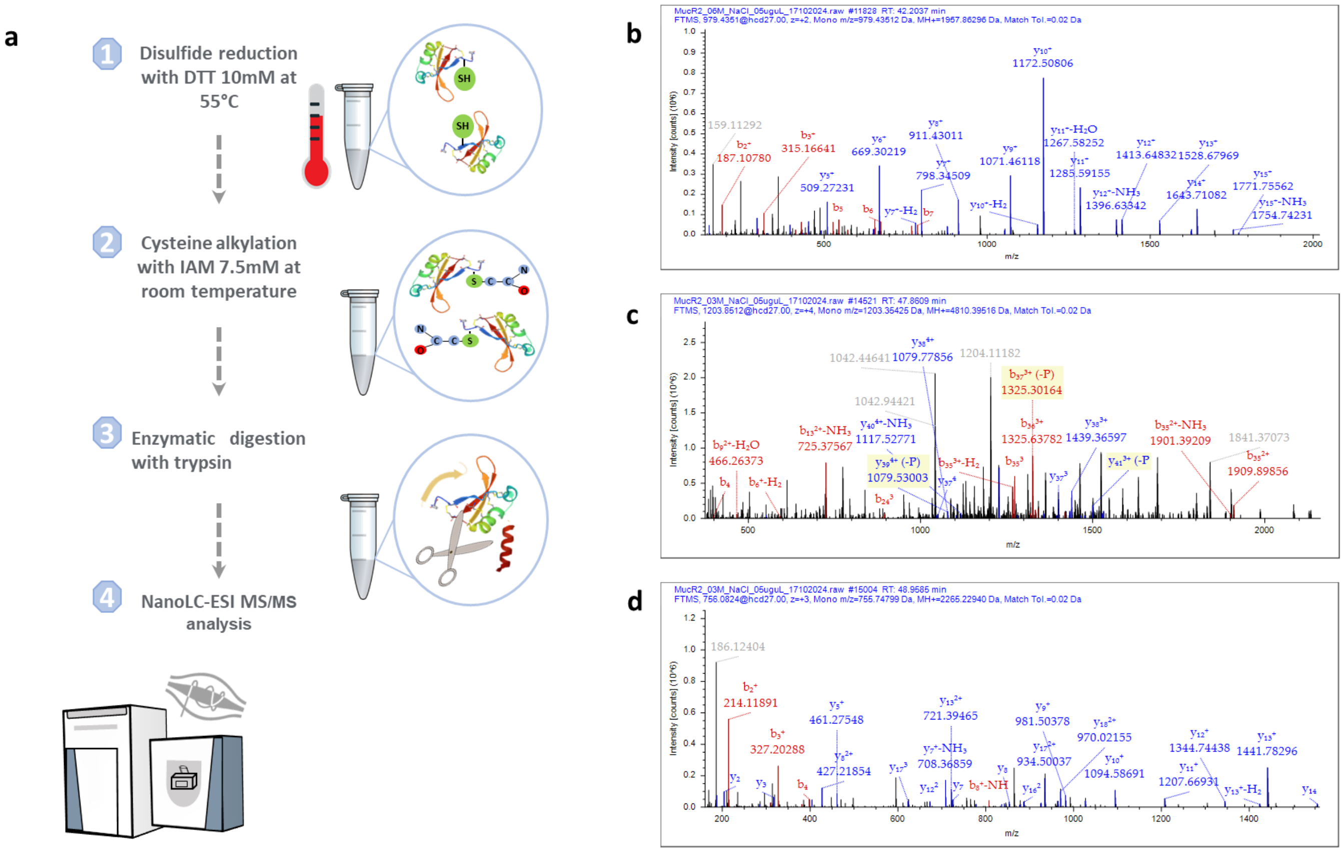

Supplementary Figure S1. a) Schematic overview of the experimental workflow used for the peptide mapping of MucR1, MucR2 and MucR3 from *S. meliloti* by using high-resolution nanoLC-MS/MS. b) MS/MS fragmentation spectrum of the doubly charged ion at m/z 979.435, mapped on the tryptic peptide 93-SVQDDQITCLECGGTFK-109 of MucR1 (AC: P55323), theoretical monoisotopic [M+H+]$^+$ mass 1597.863 Da, $\Delta = 0.02$ ppm. c) MS/MS fragmentation spectrum of the quadruply charged ion at m/z 1203.851 mapped on the tryptic peptide 50-NVIAPDELPYLIQQTYGSLNETSGPGEYPPAVEEQRPAVPIKK-107 of MucR2 (AC: O33682), theoretical monoisotopic [M+H+]$^+$ mass 4810.386 Da, $\Delta = 1.84$ ppm. d) MS/MS fragmentation spectrum of the triple charged ion at m/z 1203.851 mapped on the tryptic peptide 50-NVIAPSELPHLIQQTYGSLGK-70 of MucR3 (AC: Q92ZQ1), theoretical monoisotopic [M+H+]$^+$ mass 2265.223 Da, $\Delta = 2.49$ ppm.

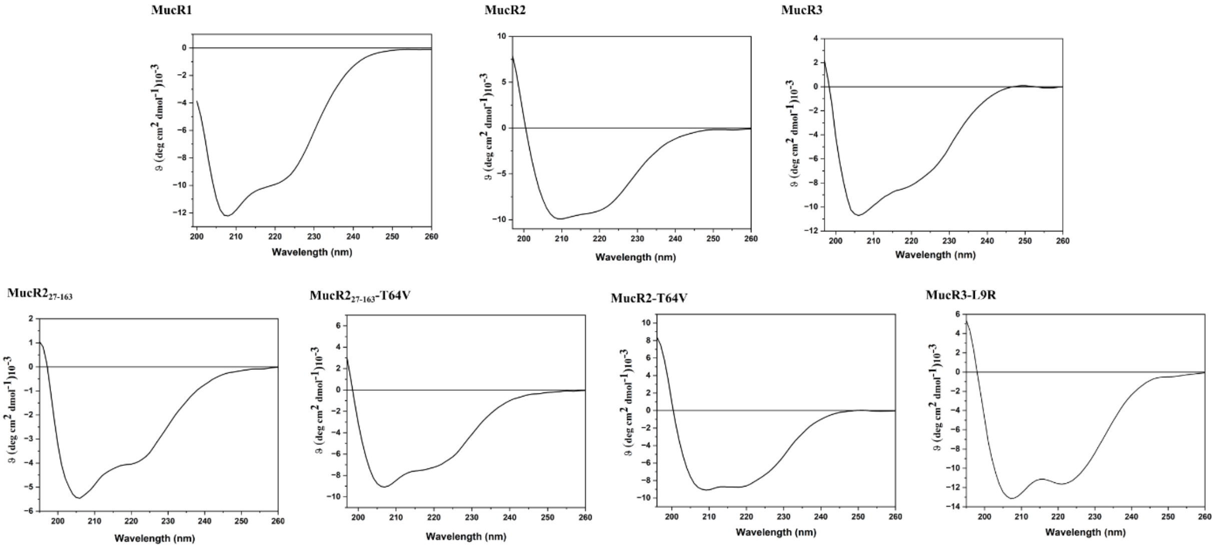

Supplementary Figure S2. CD spectra of all the proteins purified in this study. The name of the proteins is reported in figure.

MucR2		
**Protein amount**	**Bound DNA**	**Free DNA**
10 pmol	18.46 %	81.54 %
20 pmol	34.44 %	65.56 %
40 pmol	56.22 %	42.78 %
**MucR1**		
**Protein amount**	**Bound DNA**	**Free DNA**
2 pmol	28.60 %	71.40 %
4 pmol	56.45 %	43.55 %
8 pmol	79.21 %	20.79 %
10 pmol	91.37 %	8.63 %
**MucR3**		
**Protein amount**	**Bound DNA**	**Free DNA**
10 pmol	6.02 %	93.98 %
20 pmol	12.44 %	87.56 %
40 pmol	18.25 %	81.75 %

Supplementary Table S2: Analysis of EMSAs results. The percentage of buond and free DNA are reported per each amount of the proteins used in EMSA with rem site3 DNA target.

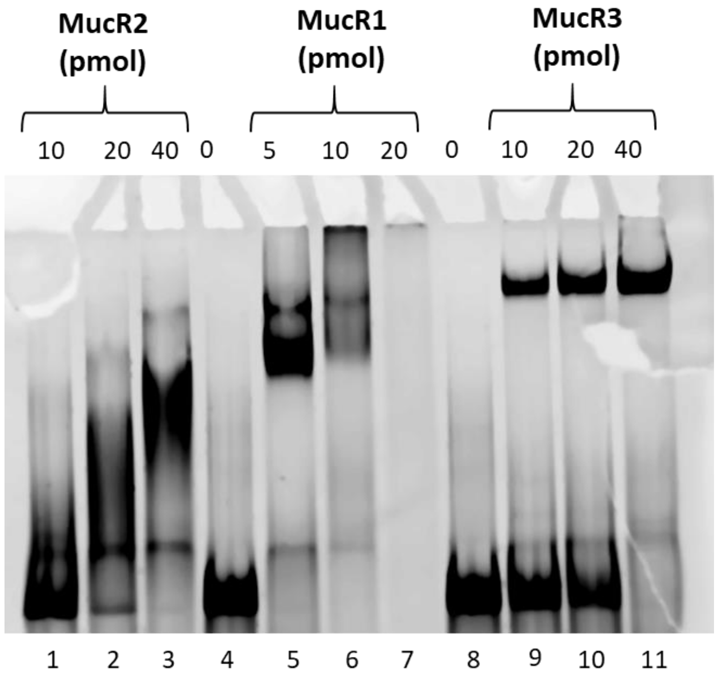

Supplementary Figure S3. EMSA with MucR1, mucR2 and MucR3 using *ndva* double-stranded oligonucleotide (Slapakova et al., 2023) [39] as target. Protein amount used are reported at the top of the lanes.

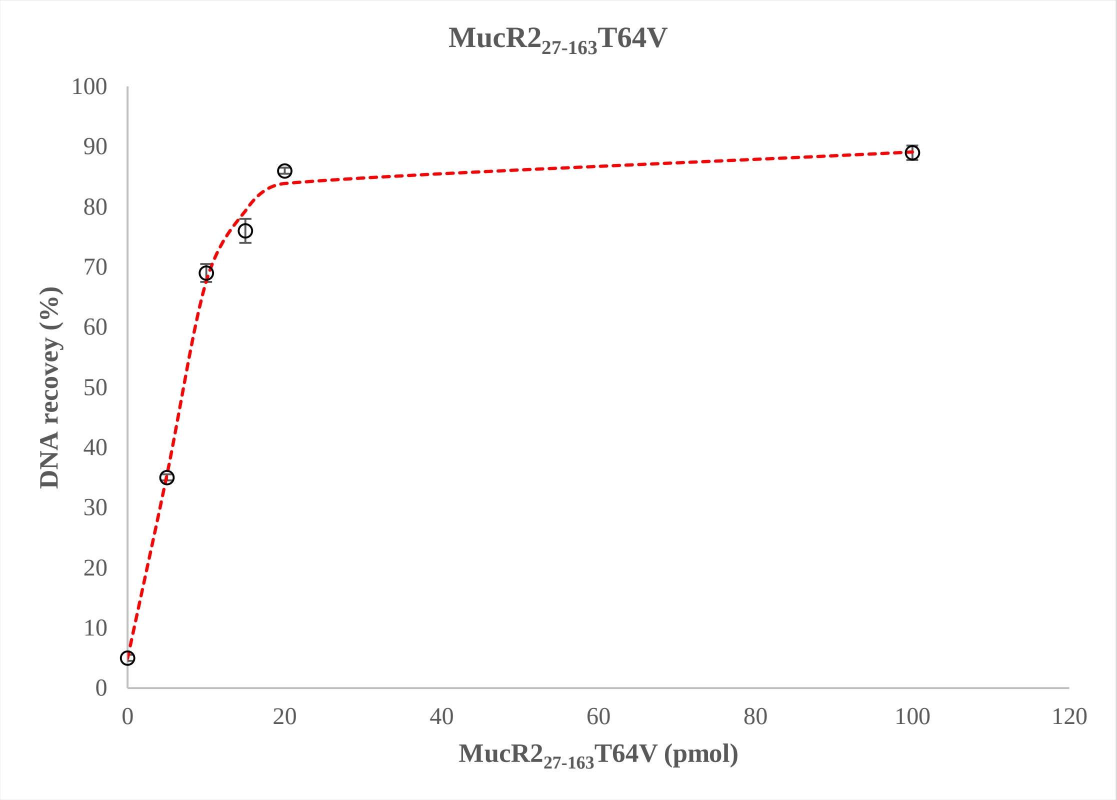

Supplementary Figure S4. Bridging assay with MucR2$_{27\text{-}163}$T64V and ndva DNA target. The amounts of protein used are indicated on the X axis, the percentage of DNA recovered on the Y axis.
